# Whole-Genome Sequence of *Monascus purpureus* GB-01, an Industrial Strain for Food Colorant Production

**DOI:** 10.1128/MRA.00196-19

**Published:** 2019-06-13

**Authors:** Toshitaka Kumagai, Masatoshi Tsukahara, Naoya Katayama, Katsuro Yaoi, Sachiyo Aburatani, Kohji Ohdan, Kazuhiro E. Fujimori

**Affiliations:** a Fermlab, Inc., Tokyo, Japan; b Biojet Company Ltd., Okinawa, Japan; c Biochemical Research Laboratory of Ezaki Glico Co. Ltd., Osaka, Japan; d Bioproduction Research Institute (BPRI), National Institute of Advanced Industrial Science and Technology (AIST), Ibaraki, Japan; e Biotechnology Research Institute for Drug Discovery (BRD), National Institute of Advanced Industrial Science and Technology (AIST), Tokyo, Japan; f Computational Bio Big Data Open Innovation Lab (CBBD-OIL), National Institute of Advanced Industrial Science and Technology (AIST), Tokyo, Japan; University of California, Riverside

## Abstract

We report the draft genome sequence of Monascus purpureus GB-01, an industrial strain used as a food colorant. *De novo* assembly of long reads resulted in 121 chromosomal contigs and 1 mitochondrial contig, and sequencing errors were corrected by paired-end short reads. This genome sequence will provide useful information for azaphilone pigments and mycotoxin citrinin biosynthesis.

## ANNOUNCEMENT

Utilization of the filamentous fungus Monascus purpureus (phylum Ascomycota) began more than 2,000 years ago for the production of red mold rice, a fermented food in Southeast Asian countries (1). Currently, it is widely used for production of yellow and red azaphilone pigments as food colorants. Recently, several research groups began conducting genetic and genomic studies of Monascus ruber (2, 3), Monascus aurantiacus (4), and Monascus purpureus (5, 6) using modern approaches to elucidate the biosynthesis of pigments and the mycotoxin citrinin. In addition, the first genome sequence of *M. purpureus* YY-1 was reported recently (7).

In this study, we obtained the *M. purpureus* strain GB-01 from Ezaki Glico Co., where the strain was collected half a century ago for the purpose of red pigment production. We selected GB-01 as a representative strain with high pigment production from among the stocks available at this company. *M. purpureus* GB-01 cells were grown in PSD100 medium (100 g/liter d-glucose, 38 g/liter polypeptone, 1.0 g/liter MgSO_4_·7 H_2_O, and 2.0 g/liter NaNO_3_ [pH 5.5]). at 33.5°C for 4 days. The genomic DNA of *M. purpureus* GB-01 was isolated using an Isoplant II kit for short reads and a NucleoSpin plant II kit for long reads according to the manufacturer’s instructions. Paired-end short reads were generated on the Illumina MiSeq platform using the Nextera DNA library preparation kit to generate libraries with different insert lengths and the MiSeq reagent kit 3 for sequencing runs. The numbers of reads totaled ∼4.00 million and 4.67 million, and their mean insert lengths were 449 and 784 bases, respectively. Long reads of *M. purpureus* GB-01 were generated on the PacBio RS II platform using the PacBio SMRTbell template prep kit 1.0 and PacBio DNA/polymerase binding kit P6. The total number of subreads was ∼1.23 million, the total number of bases was ∼2,862 million, the mean subread length was ∼2,318, bases and the *N*_50_ subread length was ∼2,536 bases. To extract the mitochondrial reads, all long reads were mapped to the Aspergillus nidulans FGSC A4 complete mitochondrial genome sequence (GenBank accession number JQ435097) using Minialign 0.5.3 (8). The reads that did not map to the mitochondrial genome were processed as chromosomal reads and assembled *de novo* using Canu 1.7 (9). The assembly errors were corrected with the PacBio genomic consensus tool using the Arrow algorithm (Pacific Biosciences). In addition, to eliminate small indels, a final polish of the assembly was performed using Pilon 1.22 with nonredundant short paired-end reads of approximately 208.7× total coverage (10, 11). In the final assembly, we obtained 121 chromosomal contigs with 24.3 million total bases with an *N*_50_ value of 327,944 bases and 1 circular mitochondrial contig with 27,264 bases.

Obtaining multiple genomic sequences from *Monascus* spp. will help establish the molecular machineries for pigment and fungal toxin biosynthesis and may lead to the development of engineered strains with improved pigment productivity and lower mycotoxin levels.

### Data availability.

The draft genome sequence of GB-01 was deposited in DDBJ/GenBank under accession numbers BIYA00000000 for chromosomes and AP019407 for mitochondria, SRA accession number DRA007939, and BioProject number PRJDB7887.
